# Quantifying Vocal Mimicry in the Greater Racket-Tailed Drongo: A Comparison of Automated Methods and Human Assessment

**DOI:** 10.1371/journal.pone.0089540

**Published:** 2014-03-06

**Authors:** Samira Agnihotri, P. V. D. S. Sundeep, Chandra Sekhar Seelamantula, Rohini Balakrishnan

**Affiliations:** 1 Centre for Ecological Sciences, Indian Institute of Science, Bangalore, India; 2 Department of Electrical Engineering, Indian Institute of Science, Bangalore, India; Claremont Colleges, United States of America

## Abstract

Objective identification and description of mimicked calls is a primary component of any study on avian vocal mimicry but few studies have adopted a quantitative approach. We used spectral feature representations commonly used in human speech analysis in combination with various distance metrics to distinguish between mimicked and non-mimicked calls of the greater racket-tailed drongo, *Dicrurus paradiseus* and cross-validated the results with human assessment of spectral similarity. We found that the automated method and human subjects performed similarly in terms of the overall number of correct matches of mimicked calls to putative model calls. However, the two methods also misclassified different subsets of calls and we achieved a maximum accuracy of ninety five per cent only when we combined the results of both the methods. This study is the first to use Mel-frequency Cepstral Coefficients and Relative Spectral Amplitude - filtered Linear Predictive Coding coefficients to quantify vocal mimicry. Our findings also suggest that in spite of several advances in automated methods of song analysis, corresponding cross-validation by humans remains essential.

## Introduction

The fundamental step to any study of vocal mimicry is to distinguish between mimicked calls and species-specific calls in an objective manner. This is usually done by listening to available sound libraries of a number of different species and identifying model species based on human psychophysical, often qualitative, perceptions of similarities between calls. This is commonly backed by visual inspection of spectrograms [1]–[3]. This method is qualitative and suffers from listener bias. In addition, sound libraries rarely include the repertoires of the putative models that a mimic might hear in the wild.

A more quantitative way of defining mimicry is to compare spectral features of the mimicked calls with those of the putative model calls using various statistical measures such as Multivariate Analysis of Variance [4], Multitaper Spectral Analysis [5], Discriminant Function Analysis [6], [7], Spectral Cross-Correlation [8]–[11], and Principal Component Analysis [12]. Tchernichovski et al. [5] and Cortopassi and Bradbury [8] examined vocal imitation, i.e. vocal similarity between individuals of the same species. Putland et al. [6] and Flower [4] quantified the mimicry of a single model species by their respective mimics (Albert's lyrebirds, *Menura alberti* and fork-tailed drongos, *Dicrurus adsimilis*, respectively). Two other studies tested the role of female choice as a driver for mimetic accuracy and examined vocal resemblance between the mimic and two model species each [7], [9]. Hamao and Eda-Fujiwara [12] were, however, probably the first to attempt an objective definition of mimicry in their study of the black-browed reed warbler (*Acrocephalus bistrigiceps*). They were also the first to study mimicry of a relatively large number of model species (eight).

Mimicry, by definition, implies call similarity, both structural and perceptual, since perceptual similarity must have a structural basis. It is therefore important to assess structural similarity between calls in studies of vocal mimicry. In our study we consider a call to be a mimicked one if it is more similar to the call of a model species than to: i) other calls of its own species and (ii) calls of a large number of other species with whom it shares the same habitat. Recently, Igic and Magrath [11] have used similar criteria to establish vocal mimicry in the brown thornbill (*Acanthiza pusilla*). They used a combination of frequency measurements from spectrograms and spectral cross-correlation to examine similarity between brown thornbill calls and those of five different model species and cross-validated the results by human inspection of call spectrograms.

In both birds and humans, sounds are produced during expiration by the flow of air through the vocal system. Even though the vocal organ in birds is structurally distinct from that of humans, acoustic output in both is produced by the ‘source-filter’ model [13]. Speech processing and recognition methods are thus increasingly being used in the automatic recognition of bird calls [14]–[16]. However, none of these studies have examined the utility of feature extraction methods used in human speech for the definition and quantification of vocal similarity between a non-human vocal mimic and its models.

The greater racket-tailed drongo, *Dicrurus paradiseus*, is well known for its ability to imitate other species [17], but there are only a few comprehensive studies on its mimicking behaviour [1], [18]. In this paper, we have attempted to quantify vocal mimicry by the greater racket-tailed drongo using spectral feature extraction methods employed in speech processing coupled with different similarity measures. We compare the results of the automated methods with human assessment of similarity based on visual examination of spectrograms.

## Methods

### Recordings

All recordings of the greater racket-tailed drongo and putative model species were made at the Biligiri Rangaswamy Temple Tiger Reserve (77°–77°16′E, and 11°47′–12°09′N), a 540 sq. km area of forests at the junction of the Western and Eastern Ghats in southern India. Audio recordings were made at a sampling rate of 48 kHz using a portable Marantz PMD 671 digital recorder and a Sennheiser ME 66 directional microphone. Spectrograms of the recordings were generated in RAVEN [19], using a Hann window, a window length of 256 sampling points and a time grid overlap of 50 per cent.

**Figure 1:**
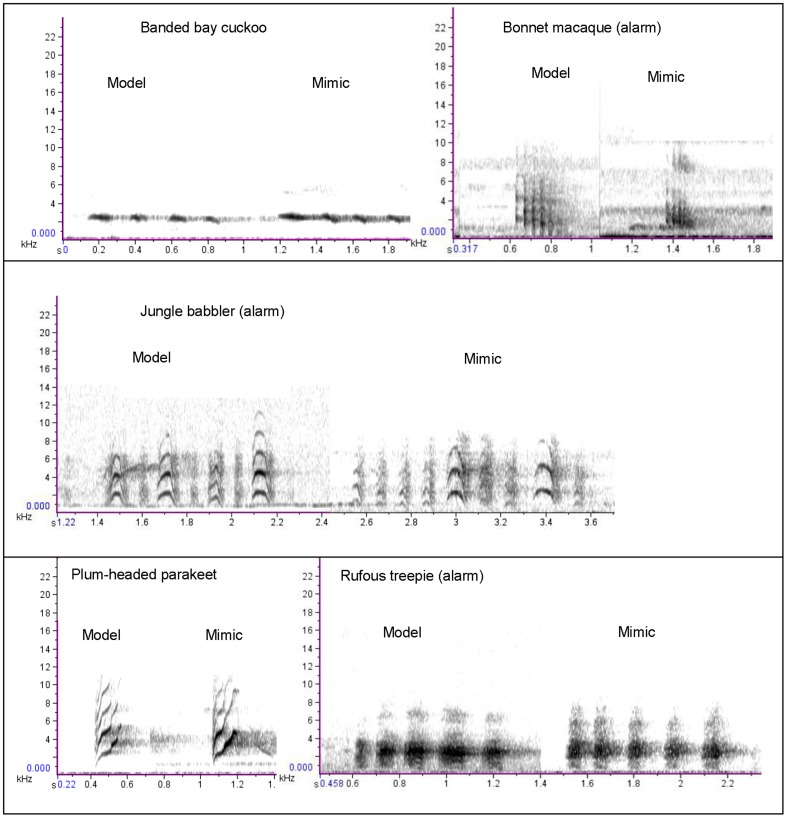
Model and mimic call spectrograms for five species (four birds and one mammal).

Samira Agnihotri (SA) has worked on the calls and songs of bird species in this area for eight years and identified mimicked calls in the recordings as a trained listener of bird calls. The phrase ‘mimicked call’ thus refers to notes/calls in the racket-tailed drongo's repertoire classified as mimicked based on the aural and visual perception of a highly trained listener (SA). This classification is used as the reference against which other classification results are compared in this manuscript. Examples of mimicked calls as classified by SA are shown in Figure 1.

All necessary permits were obtained for the described field studies from the Karnataka State Forest Department.

### Human assessment

We asked 105 volunteers to assess similarity between mimicked and model calls by showing them spectrogram images of the same. Typically, cross-validation by humans involves psychophysical methods where one or more individuals are first trained to recognise and distinguish between sounds or are already familiar with them, and then asked to identify similarity by listening to a range of sounds in an experimental setup. We believe that inter-individual variability is high in untrained listeners and tried to reduce this by opting for visual inspection of spectrograms, which are accurate time-frequency representations of the time-varying audio components of such sounds.

We created spectrogram libraries of four types:

- Mimicked calls - (21)
- Putative heterospecific model calls (“models”) - (21);
- Putative heterospecific non-model calls (“other species”) - (20)
- Racket-tailed drongo putative non-mimicked calls (“species-specific”) - (20)

Spectrograms of the sounds in these libraries were saved as JPEG files at a uniform resolution (3.75 s/line; 24 kHz/line) and numbers on the axes were removed digitally. We randomly picked 3 notes each from the “mimicked calls” (see Table 1 for the contents of this library), “other species” and “species-specific” libraries to create 105 sets of 9 notes each. We then added a mimicked call and its putative model to every set such that each mimicked call was represented in five sets. Thus, we had a total of 105 sets of 11 spectrograms each. All sets were shuffled and their contents numbered sequentially in order to remove any biases in the arrangement of the spectrogram images.

**Table 1:** Putative models and call types mimicked by the greater racket-tailed drongo.

Species		Call type	Spectral signature
*Birds*			
Banded bay cuckoo	*Cacomantis sonneratii*	Call	FM
Black-rumped flameback	*Dinopium benghalense*	Call	Trill
Common hawk-cuckoo*	*Hierococcyx varius*	Call	FM
Common tailorbird*	*Orthotomus sutorius*	Call	FM
Crested serpent eagle	*Spilornis cheela*	Call	FM
Crested treeswift	*Hemiprocne coronata*	Call	HR
Green bee-eater#	*Merops orientalis*	Call	Trill
Jungle babbler	*Turdoides striata*	Alarm	HR
Large billed crow	*Corvus macrorhynchos*	Call	NB-Trill
Loten's sunbird#	*Cinnyris lotenius*	Call	BB
Oriental honey buzzard*	*Pernis ptilorhyncus*	Call	FM
Oriental honey buzzard* #	*Pernis ptilorhyncus*	Courtship	FM
Oriental white-eye	*Zosterops palpebrosus*	Call	NB-Trill
Plum-headed parakeet	*Psittacula cyanocephala*	Call	HR
Red spurfowl#	*Galloperdix spadicea*	Call	HR
Rufous treepie	*Dendrocitta vagabunda*	Call	FM
Rufous treepie	*Dendrocitta vagabunda*	Alarm	BB
Shikra*	*Accipiter badius*	Call	HR
White-breasted kingfisher	*Halcyon smyrnensis*	Call	Trill
Yellow-browed bulbul	*Iole indica*	Call	FM
*Mammals*			
Bonnet macaque	*Macaca radiata*	Alarm	HR

# - files that were misclassified in the human assessment.

*- files that were misclassified by the computer-based method.

During the experiment, a person was shown the test spectrogram (‘mimicked’) and asked to identify and rank the two most similar spectrograms from the remaining 10 in the set. Each set was shown to a different person. All subjects were students from electrical engineering and biology departments who were familiar with signal processing methods and spectrograms but naive to the purpose of this study. No time limit was set for the task, but most individuals completed within five minutes.

All participants gave informed verbal consent to participate in the study. Their names and institutional affiliations were recorded with their consent but are kept confidential. Approval for this component of the study, including the consent procedure, was obtained from the Institutional Human Ethics Committee of the Indian Institute of Science, Bangalore (IHEC No. 16/2013).

### Computer-based methods

We used the following methods commonly used in human speech analysis to extract spectral feature vectors and calculate similarity between mimicked and putative model calls.

**Figure 2:**
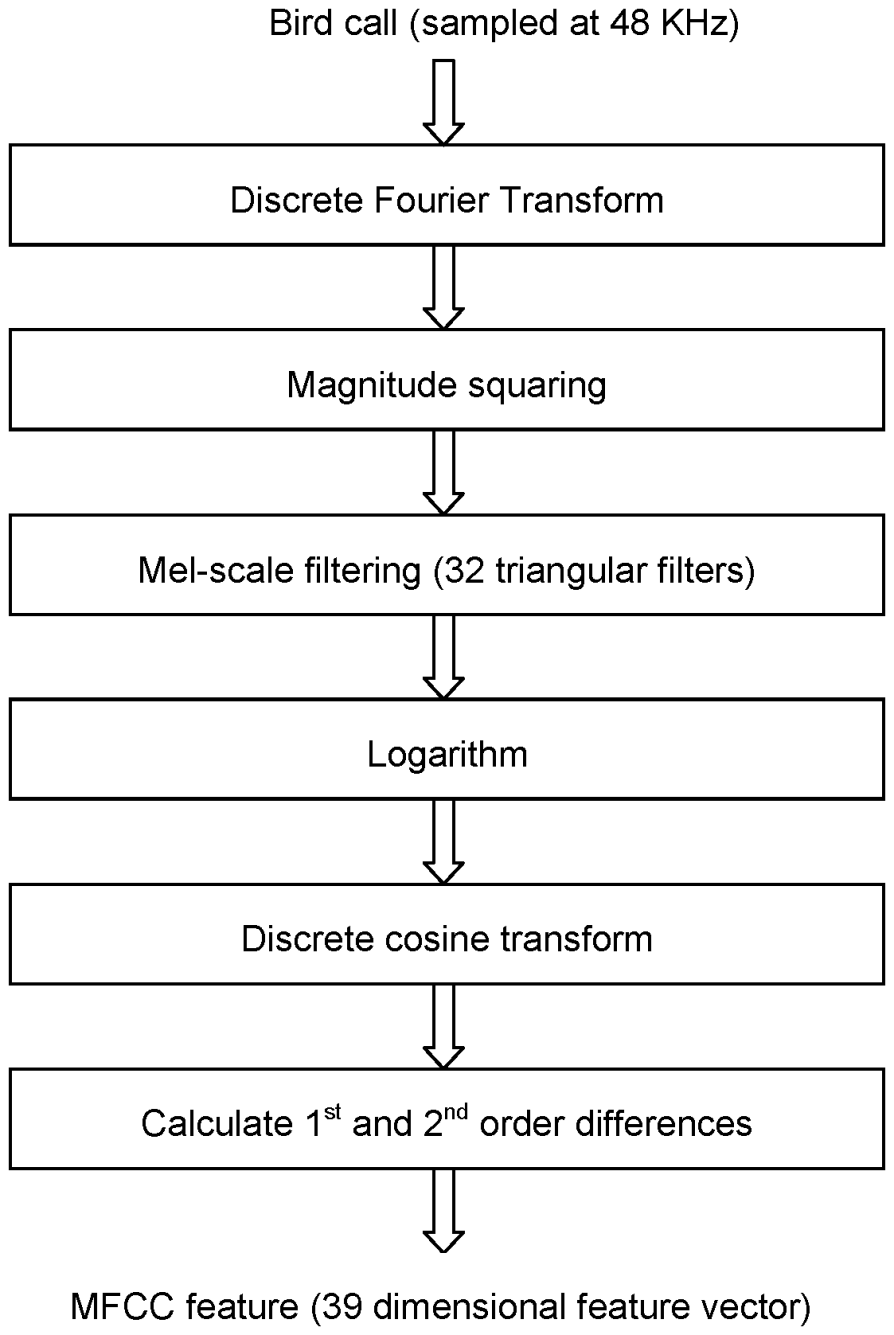
Flow chart showing the procedure for computation of mel-frequency cepstral coefficients.

The critical aspect in a feature vector derivation is that it should be a smooth representation of the underlying short-time spectrum of the signal. Some important representations that have become successful in speech recognition, which are relevant to the problem at hand are the following:

- *Mel-Frequency Cepstral Coefficients* (MFCC), [20]: The procedure for extracting short-time MFCC features is shown in Figure 2. The sampling frequency of the input call is 48 kHz. A Hamming window of duration 25 milliseconds is used in the computation of the short-time spectrum. This corresponds to a sample size of 1200. The overlap between consecutive frames is 480 samples. The short-time spectrum of each frame is computed and the spectral magnitudes are squared to obtain the corresponding power spectrum. The spectrum is then averaged on the mel-scale using triangular filters. The result is an array of short-time energies, one per band. The energies are then subject to a logarithmic transformation, which essentially performs dynamic range compression in agreement with intensity compression performed in the auditory system. The 32 log energies constitute a smooth representation of the spectrum, with high resolution at low frequencies and low resolution at high frequencies. The variable auditory resolution is based on psychoacoustic masking experiments carried out on human subjects. Knudsen and Gentner [21] showed that songbird audition is in many ways similar to human audition. Specifically, the properties related to frequency range, spectral sensitivity, temporal sensitivity and masking are quite similar across songbirds and humans, although there are minor differences in the detection thresholds. Based on these findings, we infer that the MFCC method, which is primarily based on the properties of human audition, should also be well suited for bird audition.
  The log power spectrum thus obtained is subject to a discrete cosine transform (DCT), which results in what is known as the mel cepstrum. Typically, the first few coefficients are significant and following speech/speaker recognition experiments, the first 13 coefficients are used to constitute a mel-frequency cepstral coefficient (MFCC) feature vector. In addition to the *static* coefficients, dynamic velocity and acceleration parameters [22] are also computed and appended to form a 39 dimensional feature vector. The 39 dimensional vector is used for matching calls. The MFCC computations in this paper were made by using the Voicebox toolbox software available at http://www.ee.ic.ac.uk/hp/staff/dmb/voicebox/voicebox.html
- *Line spectral frequencies* (LSF), [23]: The LSF parameterization is a discrete representation of the spectrum in the sense that it is a collection of spectral indices about the short-time spectral resonances. LSFs have been shown to be robust to quantization, which makes them a natural choice for compression applications [24]. To start with, one considers the linear prediction (LP) model [25] of a random signal 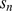, and constructs an approximation 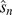 based on the past 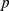 samples of 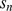 as follows in equation (1):
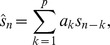
where the coefficients 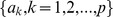 are optimized such that 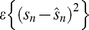 is minimized, where 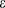 denotes the ensemble averaging (expectation) operator. This yields a standard set of Yule-Walker equations (or normal equations, which are linear in the unknown parameters) and the coefficients can be obtained by solving them using Levinson-Durbin recursion [23]. The LP model essentially constitutes an autoregressive or all-pole model of the short-time spectrum on a linear magnitude scale. From the LP polynomial 
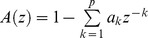
 one constructs two polynomials 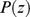 and 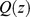 such that in the following equations (2):
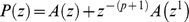
and (3):
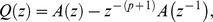
which, in case of human speech, correspond to the vocal tract configurations with glottis closed and open, respectively. Interestingly, 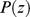 is a palindromic polynomial, and 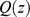 is anti-palindromic. The roots of 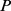 and 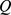 lie on the unit circle in the complex plane and they also mutually alternate. The roots also occur in complex conjugate pairs. The locations of the roots of 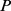 and 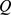 constitute the LSF representation for a short-time signal segment.
- *RASTA-PLPCC features*: The standard linear prediction model results in a smoothed short-time spectral envelope. The main feature of the LP model is that it approximates the short-time spectrum equally well at all frequencies that fall within the spectral band. While this feature may be viewed as a merit from a generic spectral estimation approach, from the perspective of auditory perception by humans (which is the benchmark for cross validating the mimicry comparisons given by the automated techniques reported in this paper), it is important to have higher resolution at low frequencies and lower resolution at high frequencies. Again, since auditory perception in birds is similar to that of humans [21], the PLPCC methodology is appropriate for such a comparative approach. In perceptual linear prediction (PLP), Hermansky [26] proposed to compute an auditory-like spectrum of speech prior to approximations by an all-pole model.

**Figure 3:**
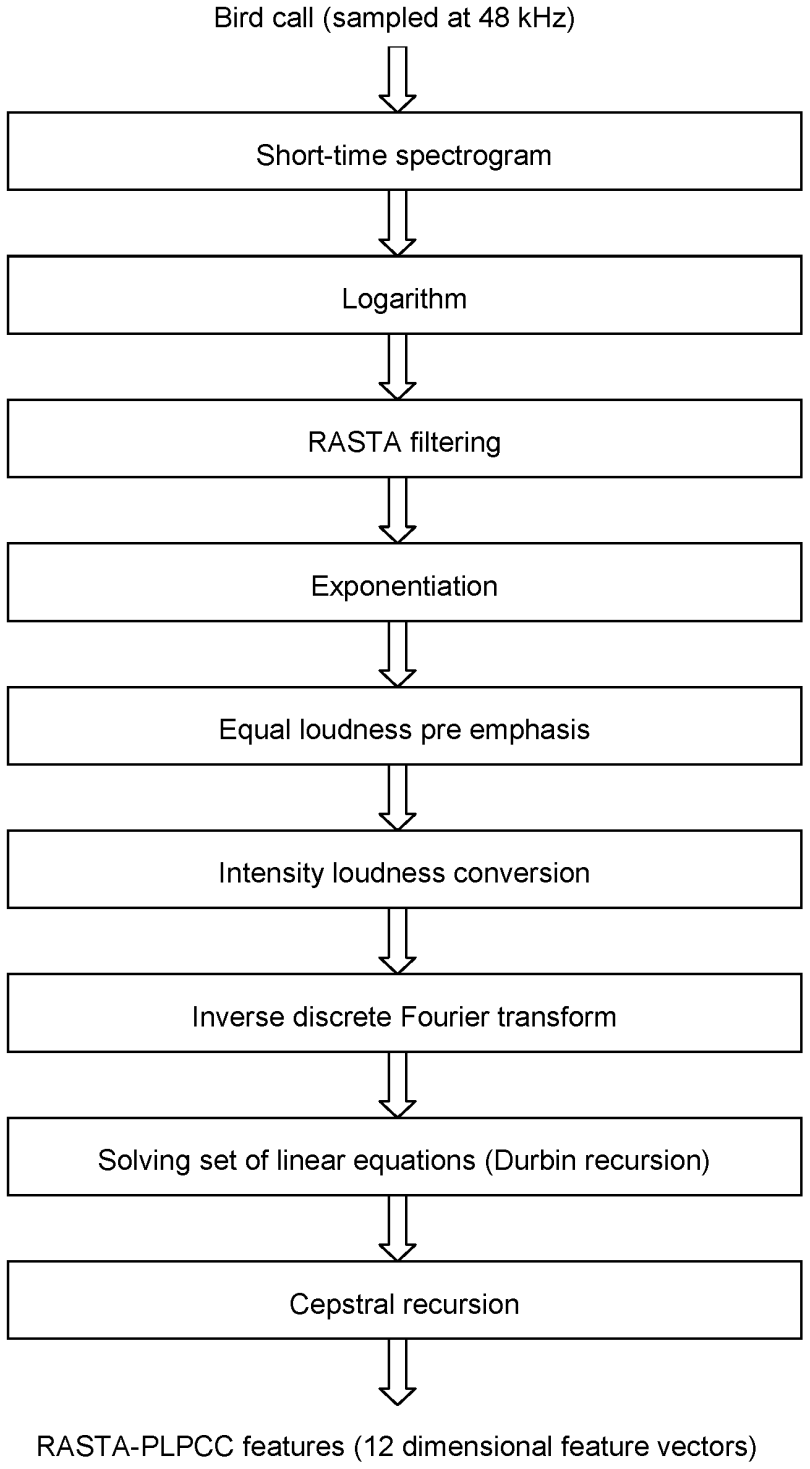
Flow chart showing the procedure for extraction of RASTA-PLPCC feature vectors.

The key steps involved in PLP are critical band smoothing of the short-time spectrum, resampling the smoothed spectrum at approximately 1-Bark intervals, pre-emphasis by an equal-loudness compensation and compression of the resulting spectrum to simulate intensity-loudness power law. The resulting spectrum has been shown to be consistent with many known results in acoustic signal perception. A feature vector parametrization of the spectrum is obtained by all-pole modeling (Levinson-Durbin recursion) and subsequent conversion to cepstral domain [22] to yield a feature vector. The cepstral coefficients (CC) thus derived are also referred to as the PLPCC. In RASTA-PLPCC [27], there is an additional filtering step on the bandpass filter outputs. The so-called RASTA filter has a sharp spectral zero at zero frequency. Consequently, constant or slowly-varying components in each sub-band output are suppressed by this operation rendering the resulting feature representation more robust to slow variations in the short-time spectrum (for example, linear distortions caused by a microphone/recording device). This operation corresponds to an implicit blind deconvolution. A block diagram representation of RASTA-PLPCC computation is shown in Figure 3. RASTA-PLPCC have been shown to be quite robust to channel distortions/mismatch and degradation in the presence of additive noise. The frame size used for short-time analysis is 25 milliseconds (1200 samples at 48 kHz sampling rate), with an overlap of 10 milliseconds (480 samples). The resulting feature vector is 12 dimensional. The feature vector computations were made using the software available at http://labrosa.ee.columbia.edu/matlab/rastamat/

We used these feature vectors in combination with the following distance metrics to calculate similarity between mimicked and putative model calls:

- *Jaccard's metric*: The Jaccard metric is a similarity measure to quantify the diversity and similarity of two given sample sets. The Jaccard distance between two sets A and B is defined as in equation (4):
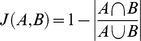
It satisfies the properties of non-negativity, symmetry, and triangle inequality.
- *Correlation coefficient*: The correlation coefficient between two vectors 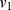 and 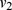 is given as in equation (5):
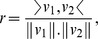
where 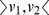 denotes the dot product of the vectors and 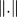 denotes the norm of the vector. The maximum value of 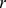 is +1 (perfect correlation) and minimum value is -1 (perfect anticorrelation).
- *Angular similarity metric*: The cosine metric is essentially the correlation coefficient itself, but one could compare two vectors solely on the basis of the angle between them. The arc cosine nonlinear transformation gives rise to the angular similarity: 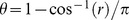.
- *ℓ_p_ distance*: The ℓ_p_ distance between two vectors v_1_ and v_2_ is defined as in equation (6):
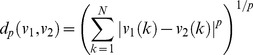
If p = 1, we have the city block metric, and p = 2 gives rise to the Euclidean distance metric.

**Figure 4:**
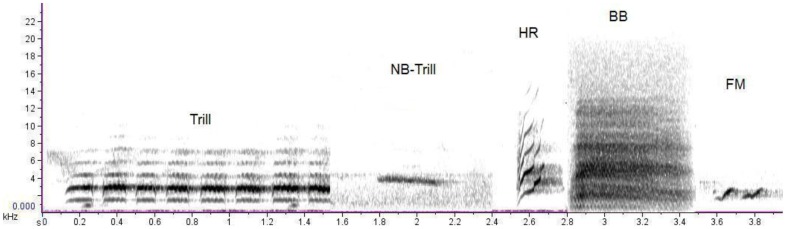
Spectral signatures of five broad classes of bird calls. Trill (Black rumped flameback, *Dinopium benghalense*); Narrowband-Trill (Oriental white eye *Zosterops palpebrosus*); Harmonic (Plum-headed parakeet *Psittacula cyanocephala*); Broadband (Black-hooded oriole *Oriolus xanthornus*); and Frequency Modulated (Yellow browed bulbul *Iole indica*).

We first created a library of 357 sound files that were segmented to include only one representative of each call. These included 63 mimicked calls, 84 heterospecific calls (including putative models) of 61 species and 210 racket-tailed drongo putative non-mimicked species-specific calls. Frequencies of the calls in these files ranged from 500 Hz to 8 kHz, spanning most of the frequency range of bird vocalisations. The notes were broadly classified on the basis of their spectral signatures (Figure 4), i.e. the shape of the spectrogram, as Frequency Modulated (FM), Broadband (BB), Harmonic (HR), Repeated trills (Trill) and Narrowband trills (NB-Trill). This was done in order to examine if the spectral feature extraction methods that we used worked better for certain types of calls (Brandes 2008). We initially tested 27 types of mimicked calls, including one call where the putative model was a mammal. Each mimicked file was compared with the remaining 356 files in the library. All computer based analyses were performed in MATLAB version 6.5 (MathWorks, Natick, MA, U.S.A.).

We used the results from this large data set to select one spectral feature extraction method and two distance metrics that gave the maximum number of correct matches. We selected the two similarity indices based on the total number of first-ranked correct matches made by the various indices. We also examined the variance in rank assignment within the first three ranks across calls (incorrect matches were scored as 4, the lowest rank, for this calculation) for all the indices.

We then repeated the analysis using these selected methods on a smaller subset of the sound files, which was identical to the subset used for the human assessment. The design for this analysis was also identical to the design for the human assessment, i.e. each of the 21 mimicked calls was tested 5 times. Each comparison was against a randomly picked set of 10 other calls (3 “mimicked”, 3 “species specific”, and 3 “other species” plus the putative model call).

We used a 2-sample test for equality of proportions with Yates' continuity correction to compare the total proportion of correct matches obtained by each method. All statistical tests were performed in R 2.13.2 [28].

## Results

### I. Human assessment

**Figure 5:**
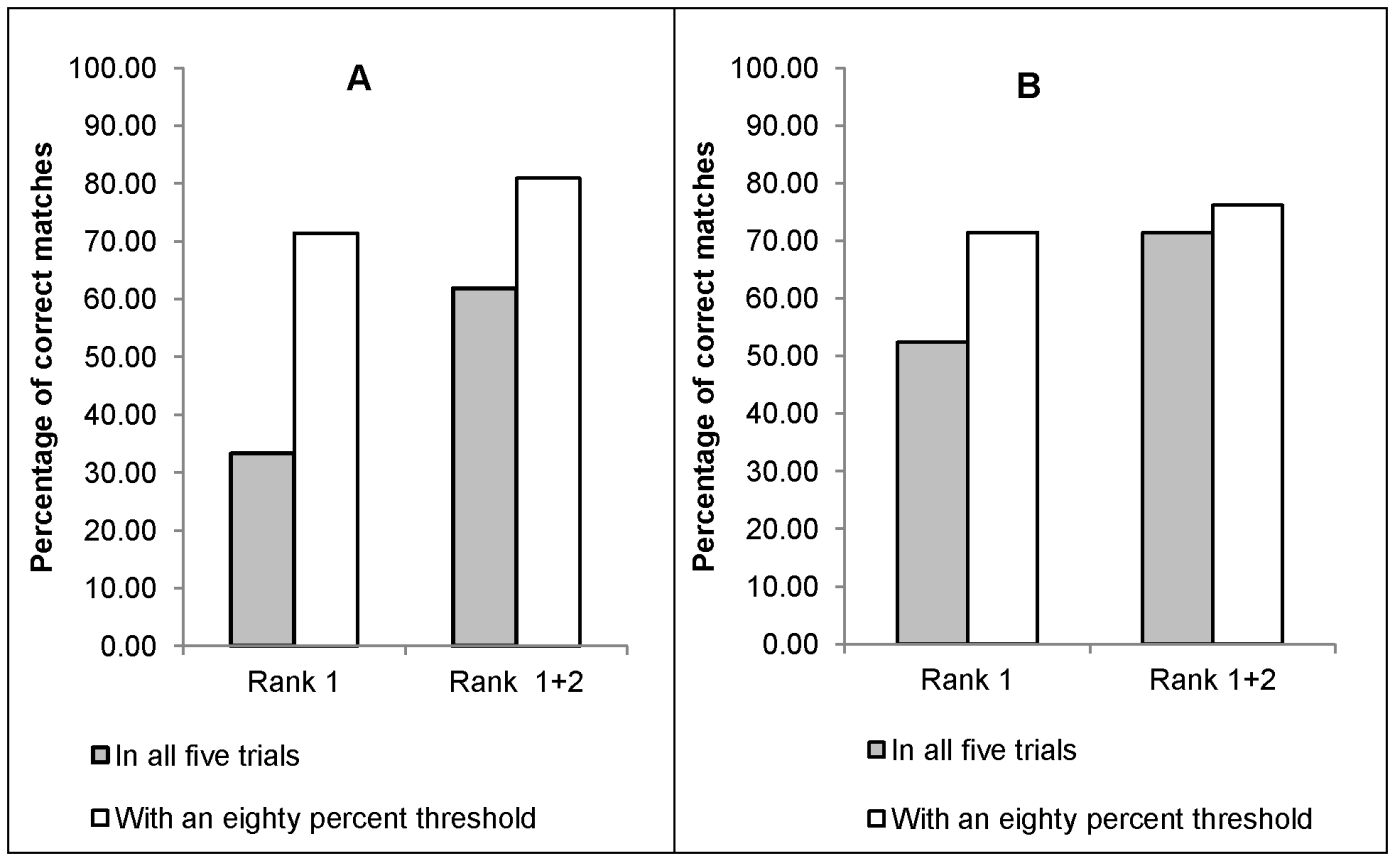
Percentage of correct matches from (A) the human assessment and (B) the computational method.

We examined how humans identified mimicry at three different levels. First, at the broadest level, 77 of the 105 people tested correctly matched a mimicked call to its model as their first choice (73.3%). This accuracy increased to 82.9% when we included second-ranked calls into the criteria for correct matches. Secondly, on a call-by-call basis, 7 out of the 21 mimicked calls tested (33.3%) were ranked as most similar to their putative model in all 5 trials (i.e. by five different people) in which they were presented (Fig. 5 A). If we included the second-ranked correct matches, the total percentage of correct matches increased to 61.9% (Fig. 5 A). At a third level, we examined the five trials for each call. If we set a threshold of 80% accuracy for each call, i.e. correct matches in at least four of the five trials per call, then according to this criterion, 15 of the 21 (71.4%) mimicked calls that were tested were matched correctly to their models in the first rank; this increased to 81% when we included second-ranked correct matches (Fig. 5 A, grey bars).

There was a significant increase in accuracy when we compared the correct matches in the first rank with and without an 80% threshold (Figure 5 A: Rank 1; *P*<0.05). This effect was not seen when the criterion for correct matches included first and second-ranked matches (Figure 5 A: Rank 1+2; *P* = 0.30). Four calls were not matched to their putative model in either the first or the second rank by human assessment (Table 1).

### II. Computer-based method

#### i. Feature vector and distance metric selection

In the first part of our analysis with a sound library of 357 files, 14 of the 27 mimicked calls (51.85%) tested showed the putative model call as the closest match (out of 356 files) across all three feature extraction methods. If we included the calls where the model was ranked as the second closest match (out of 356), the total number of files matched correctly increased to 17 (63%). The PLPCC feature vector performed best, giving 12 of these 17 correct matches. PLPCCs also performed well across three categories of call types (5/7 FM calls; all 3 BB calls and 3/4 HR calls). The Jaccard index, the correlation co-efficient and the cosine similarity indices showed the maximum number of first-ranked correct matches across feature extraction methods (Table 2). These three similarity indices also showed the most consistent results i.e. they gave identical ranks to the correct matches (Table 3, as represented by the lowest variance in rank assignment). Thus, we decided to use the PLPCC feature vector and the Jaccard and correlation indices for our comparative analysis on the subset of 82 sound files.

**Table 2:** The total number of correct matches in the first three ranks given by the five similarity indices for each spectral feature extraction method.

	RASTA-PLPCC			MFCC			LSF		
	Rank 1	Rank 2	Rank 3	Rank 1	Rank 2	Rank 3	Rank 1	Rank 2	Rank 3
**Jaccard's distance**	9	3	2	5	1	1	5	1	-
**Correlation coefficient**	9	3	2	5	1	1	5	1	-
**Cosine metric**	9	3	2	5	1	1	5	1	-
**City Block distance**	9	2	2	2	1	1	3	1	2
**Euclidean distance**	10	3	2	2	1	-	2	2	-

**Table 3:** The variance of the ranks for all correct matches within the first three ranks by the five similarity indices for the three spectral feature extraction methods.

	RASTA-PLPCC (14)	MFCC (7)	LSF (7)
**Jaccard's distance**	0.58	0.62	1.29
**Correlation coefficient**	0.58	0.62	1.29
**Cosine metric**	0.58	0.62	1.29
**City Block distance**	1.02	1.9	1.48
**Euclidean distance**	0.57	2.14	1.95

Similar values indicate consistency in assigning ranks.

#### ii. Final automated analysis

When we performed the analysis on the smaller sound library, 11 of the 21 files tested (52.38%) using the PLPCC were ranked as most similar to their putative model in all 5 trials. If we included correct matches in the second rank, the total percentage of correct matches increased to 71.43%. In accordance with our analysis of the human assessment, if we kept a threshold of 80% accuracy for each call, i.e. correct matches in at least four of the five trials per call, then according to this criterion, 15 of the 21 (71.4%) mimicked notes that were tested were matched correctly to their models in the first rank and this increased to 76% when we included second ranked correct matches. The increase in the total number of correct matches with a threshold of 80% was not significant for the first rank, nor for the first and second ranks combined (*P* = 0.34).

Five mimicked calls were not matched to their putative model in either the first or the second rank by the computational algorithms (Table 1).

### III. Comparison of computer-based methods and human assessment

Both methods showed similar results in terms of the total proportion of mimicked calls that were matched correctly to their putative models. Although the automated method performed slightly better than humans, the difference was not significant (Rank 1: *P* = 0.34; Rank 2: *P* = 0.74). The total percentage of correct matches with the 80% threshold was also similar for both the methods. When we included second-ranked correct matches within this criterion, humans made a relatively higher number of correct matches, but this difference was also not statistically significant. A closer inspection of the calls misclassified by both methods revealed little overlap between these two sets (Table 1). Only one call was not matched to its putative model in the first or second rank by both the computer-based methods and humans (Table 1: Oriental honey buzzard courtship call) and will therefore be deleted from the mimicked calls data set.

## Discussion

This is the first study of vocal mimicry in the racket-tailed drongo that has attempted to quantify mimicry using an objective approach. This is also the first study in which MFCCs and PLPCCs have been applied to describe vocal mimicry in a bird species. Overall, both computer-based methods and human assessment showed similar results (in terms of proportion of calls) in matching a mimic to its model.

These results are similar to those obtained in previous studies on automatic recognition of bird calls using feature vectors used in human speech analysis to examine the calls of a relatively large number of species [16], [29]. Somervuo et al. [29] used MFCCs and time-varying sinusoidal models for the classification and identification of the songs of 14 North European passerines. In their study, the average recognition accuracy for single syllables for most species ranged from 40% to 50%. This increased when more data were supplied to the classifiers in the form of song phrases. They argued that the low recognition accuracy could be due to the large variation in bird vocalizations. They also found that time-varying sinusoidal models of the syllables gave the overall highest recognition accuracies, especially for species with a certain type of harmonic calls. They recommend the use of hierarchical classifiers based on the tonality of the song syllable to select the most appropriate feature extraction method. Cheng et al. [16] attempted the classification and identification of the calls of 10 species of birds using LPCCs and MFCCs combined with various machine-learning algorithms. Recognition accuracy varied for each species (from 50% to 100%). Support vector (SVM) machine-learning classifiers in combination with MFCCs worked best for their data, whereas LPCCs worked better with Hidden Markov Models (HMMs). HMMs and SVMs, however, require a lot of training data, which may not always be available for all the species of interest.

In our study, we did not have any training data sets and the PLPCC gave the maximum number of correct matches irrespective of the spectrogram category (FM, BB, HR, NB-Trill, and Trill) i.e., there was no structural similarity in the types of calls correctly classified by the PLPCC. The best matching performance in our study was obtained with the RASTA-PLPCC feature vectors and the performance did not vary significantly with the type of distance metric employed. MFCC and LSF feature representations performed nearly identically up to within the first two ranks and both were found to be significantly inferior compared with RASTA-PLPCC. The LSFs are a discrete representation of the spectrum and hence more sensitive to minor perturbations of the spectrum or additive noise than MFCC/PLPCC, which are smoother representations of the short-time spectrum.

It is, however, surprising to note that MFCC feature representations and dynamic features, which have become de facto standard in speech/speaker recognition applications, did not fare well in the mimicry to model call matching task. RASTA-PLPCC equipped with the Euclidean distance metric (in terms of total number of correct matches) emerged the top performer. Also, among the three feature representations considered, it is again RASTA-PLPCC that comes quite close to modelling the peripheral auditory system behaviour using suitable engineering approximations. Given that the ground truth in the call matching task was given by a trained human listener, it is probably not surprising that RASTA-PLPCC outperforms the other two representations considered.

Our results also corroborate recent work on vocal mimicry in the brown thornbill, where human identification and classification of mimicked notes did not differ from that done by computer-based methods [11]. Their results showed 97% overlap in the identification of mimicry by computer based methods and by humans. Igic and Magrath [11] were interested in alarm mimicry of five model species (only calls above a 6 kHz threshold were selected), and they had only one species-specific alarm call for comparison, which could explain why they were able to obtain such a high degree of accuracy using only spectral cross-correlation methods (SPCCs).

In this paper, we examined 21 racket-tailed drongo calls, which include mimicry of 19 different species, and compared them with 61 other calls (including the putative models, 20 non-model species calls, and 20 racket-tailed drongo species specific calls). In our study there was 81% overlap in the sounds judged as mimicry by the trained human assessor and the untrained human assessors and 76% overlap between the trained human assessor and the automated method. If we pool the results of the automated methods and the human assessment, we have a combined accuracy of 95%. This indicates that humans were able to match certain calls that the computational algorithms were unable to and highlights the fact that cross-validation by humans is still a necessary component of studies involving automated procedures for identifying and classifying sounds. This could be especially relevant when the calls vary greatly in signal-to-noise ratio, a problem that continues to be a significant hurdle in the progress of automated sound recognition in the field [30]. Any advances in these methods would benefit from a comparison between multiple trained human assessors and the application of more complex algorithms such as support vector machine-learning and neural networks, especially when we take into account the large variation in the types of calls being examined.

Quantitative methods to study vocal mimicry provide a useful tool in identifying and establishing variations in mimicry repertoires across mimicking individuals. They can also be used to examine the accuracy of imitation between models and their mimics [6], [9]–[11]. Our results reveal however that the automated methods still need to be refined and improved to obtain higher levels of accuracy, even if they are currently close to the levels of accuracy achieved by untrained human observers. Identification of mimicry based on structural features of calls is however just the first step and further exploration into its functions would require information on the contexts in which mimicry is produced, as well as bioassays involving the target (receiver) species in the wild [10].
